# A Dynamic Monitoring Method of Public Opinion Risk of Overseas Direct Investment—Based on Multifractal Situation Optimization

**DOI:** 10.3390/e25111491

**Published:** 2023-10-28

**Authors:** Yong Li

**Affiliations:** Business School, China University of Political Science and Law, Beijing 100088, China; yongli@cupl.edu.cn

**Keywords:** overseas direct investment (ODI), media-based negative sentiment, multifractal, fractal interpolation, trend prediction

## Abstract

The negative public opinions and views on overseas direct investment (ODI) of a multinational enterprise (MNE) will damage the image of its brand and are likely to bring it serious economic and social losses. So, it is important for the MNE to understand the formation and spread mechanism of public opinion risk (POR) in order to effectively respond to and guide the public opinion. This research proposed a multifractal-based situation optimization method to explore the POR evolution based on the media-based negative sentiment on China’s ODI. The sentiment measurement is obtained by a directed crawler for gathering the text of media reports corresponding to a certain ODI event using a URL knowledge base from the GDELT Event Database. Taking the public opinion crisis of the tax evasion incident of the local arm of China’s MNE in India as an example, the experiments show that this method could dynamically monitor the POR event in real-time and help MNE guide the effective control and benign evolution of public opinion of the event.

## 1. Introduction

The public opinion risk (POR) has gradually become one of the most prominent risks that multinational enterprises (MNEs) have to face in overseas direct investment (ODI) in recent years because the market environments are impacted by more complicated factors, such as a lack of consistent commitment resulting from the change in policies or regulations or change in government of the host country, as well as the conflicts caused by cross-cultural differences in human value, ways of thinking and behavior, ethnic characteristics, religious beliefs, and customs. According to our survey on 60 failed ODIs of China’s MNEs from 2014 to 2023, more than 65% of the MNEs believe that negative public opinion plays an important role in determining the failure of an ODI. Most of the failed ODIs have experience with “public opinion suspiciousness—official of the host country keeping silence—public opinion maintaining ferment—the situation getting out of control—the host country’s officials taking adverse action on the local arm of MNE”. So, it is essential for MNE to effectively detect and control POR in order to safely operate abroad.

Generally, the POR refers to the negative information, false information, and rumors that enterprises and/or organizations may face from society or media when they engage in economic activities and social management. This negative information fermentation could generate a public opinion crisis. The POR of an ODI refers to the negative social and political attitudes held by the public toward the emergence, spread, and change in ODI events in the host country. With the increasing popularity of social media platforms, all kinds of flows of information have, to some extent, shifted from the periphery to the core and PORs could easily arise for an ODI.

Combing through the existing literature, it is found that most studies on MNEs’ ODI risks focus on political risk, legal risk, and market risk, but there are few studies on the detection and control of the POR of ODI events in host countries. One reason may be due to the difficulty of quantifying PORs, the other is the difficulty of achieving an effective insight into the actual situation of public opinion of new emergencies due to the uncertain influence of environmental factors at the time. In recent years, the development of big data science and technology has provided a new method and approach to quantitatively measure the POR. The GDELT Project monitors the world’s broadcast, print, and web news from nearly every corner of every country in over 100 languages, identifies the events driving our global society, and has created a free open platform for computing the POR (https://analysis.gdeltproject.org/index.html (accessed on 25 January 2023)). People just need to simply specify a set of criteria for the event type and actors involved, along with an optional date range, and the system will automatically search the entire GDELT Event Database for all matching entries and export matching records as a CSV file.

This paper firstly collects the media-based negative sentiment comments for an ODI event as a measurement of the POR of an ODI. A negative “tone” report related to an ODI event in the media counts as −1, statistics collection is performed every one hour, all the negative scores are added and used as a measurement of the POR at that time. Gathering observations obtained through repeated measurements over time, we obtain the POR time series data. Secondly, we propose a dynamic monitoring method of the POR, based on the rich historical data of various types of POR events of an ODI from China. It includes the following two steps: The first is to construct a multifractal-based POR situation discriminative model to group the POR events correctly. The second step is to construct a fractal interpolation-based POR situation prediction model to make subsequent predictions for new POR events. Finally, taking the public opinion crisis of the tax evasion event of a local arm of China’s MNE in India as an example, we check the feasibility of the method. Experiments show that this method can monitor the POR of an ODI event in real-time and help MNEs guide the effective control and benign evolution of a POR in the process of investment and operation of overseas projects.

The remaining sections of this paper are as follows: In Section 2, we review the prevalent modeling methods of detection and early warning of a POR among academia. In Section 3, we introduce all the technical methods used in this paper, including the multifractal-based POR situation discriminative model, fractal interpolation based on iterative function system (IFS), etc. In Section 4, we present the empirical analysis of “the tax evasion event” of a local arm of China’s MNE in India and a robustness test for the fractal interpolation-based forecasting method. Section 5 concludes.

## 2. Literature Review

Some typical studies relevant for POR monitoring include the following: Wang et al. [1] presented a multidimensional and multilayered public opinion network model to effectively characterize the sentiment tendencies of multiple topics. Gao [2] proposed a comprehensive causal relationship model to explore the factors affecting network public opinion based on system dynamics. Cheng [3] analyzed the evolution tendency of public opinion and propose a structural hole-based approach to control public opinion in social networks. Zhang [4] established a three multi-topic network public opinion propagation models and used stability theory to analyze the stability of the public opinion equilibrium point of the three models. Karamouzas, et al. [5] presented an automated public opinion monitoring mechanism, which consists of semantic descriptors that relies on natural language processing algorithms to quantify general public opinion. 

In response to the urgent need for POR early warning, Zhu [6] proposed a time model of POR based on the life cycle of emergencies, time series prediction, and the natural law of public opinion evolution, which could be used for quantitatively evaluating trends in public opinion. Li and Li [7] incorporated components such as external environmental influences, individual personality characteristics, and interpersonal intimacy into a public opinion propagation model to fully reflect the law of public opinion propagation in real life. Meng et al. [8] proposed a method to rate the crisis of online public opinion based on a multi-level index system to realize the crisis warning of online public opinion. Peng et al. [9] constructed an early warning index system for network public opinion that includes 4 first-level indicators and 13 second-level indicators and illustrated its feasibility. Li et al. [10] built an early warning system of network public opinion by using big data, and then verified the system could be used to effectively identify the risks of public opinion information during the germination period.

As for the study of the spread of negative sentiments, with the development of social platforms, more and more researchers would like to use social platforms to understand people thoughts and sentiments on particular topics, and how these evolve over time. Binifazi et al. [11] proposed a new investigation on scope to assess the scope of the sentiment of a user on a topic. Yin et al. [12] proposed a framework to analyze the evolution of sentiments on COVID-19 from tweets related to this topic collected over two weeks. Xu et al. [13] concerns the dynamics of topics in the context of online news by exploiting a manifold learning-based model. Pröllochs et al. [14] focused on the analysis of the relationship between sentiment and fake news, and investigated how the spread of sentiment can cause a viral spread of fake news in social media. Gallacher and Bright [15] presented a study of the spread of hate speech in online social media. Binifazi et al. [16] presented a framework to support analyses of the dynamics of user and community sentiments in a social platform.

Although scholars have conducted a lot of research on the monitoring and early warning strategies of PORs, there are relatively few studies on the dynamic fitting of POR evolution considering real-time public participation, except for a study by Guan et al. [17], who introduced the fractal interpolation theory to predict the public opinion crisis of an electric power enterprise’s digital transformation. In particular, ODIs are facing a changing international environment full of uncertainty and instability. It is usually difficult to fit the POR trend of new ODI events well according to the existing POR evolution mode only. Therefore, it is necessary to incorporate real-time public participation information to a historical POR mode in forecasting the POR trend so as to reduce the deviation and lag of the POR prediction and achieve an effective insight into the POR situation of new ODI emergencies.  

The marginal contributions of this paper are as follows: First, it constructs a POR situation discriminative model, and explores the multifractal characteristics of various types of POR evolution based on the collected historical data of POR events of China’s ODI. Second, it constructs a fractal interpolation-based POR prediction model to fit a POR future situation. Third, taking the public opinion crisis of the tax evasion incident of the local arm of a China’s MNE in India as an example, we check the models’ validity. Experiments show that this method could dynamically monitor the POR event in real-time, and help MNEs guide the effective control and benign evolution of the POR events.

## 3. Research Design

### 3.1. Construct the Multifractal–Based POR Situation Discriminative Model

We define a framework based on Guan et al. [17] for the classification of the POR events, it involves the following three steps: The first is to cluster the POR events using mean-shift algorithms. The second step is to extract multifractal features for each class. The last step is to use multifractal features as input vector for training a support vector machine (SVM) classifier. Along with the training, the POR events with similar multifractal features are gradually grouped together, which can help us to verify the correctness of the assigned clusters by the mean-shift algorithm. This multifractal-based POR situation discriminative model will provide basic support for more accurate fitting and predicting the subsequent trend of the POR.

#### 3.1.1. Classify POR Events Using Mean-Shift Algorithms

The POR events are grouped tentatively using mean-shift algorithms. The mean-shift is a centroid-based algorithm, which works by updating candidates for centroids to be the mean of the points within a given region. These candidates are then filtered in a post-processing stage to eliminate near duplicates to form the final set of centroids. The specific process is as follows: Firstly, starting from an initial designated centroid x, each POR situation is represented as a point in a d-dimensional space, i.e., $x=(x_{1},x_{2},\ldots ,x_{d})$, and calculate the mean-shift in the given region. If the total number of POR propagation trends collected are n, each POR trend is expressed as $x_{i}$ (*i* = 1, 2, …, *n*). In order to determine the weights of x neighborhood of points, we compute the mean-shift for each centroid that points towards a region of the maximum increase in the density of points based on the Gaussian kernel function $K=(x_{i}-x)$. The density-weighted mean at point x is:

(1) $$m\left(x\right)=\frac{\sum_{x_{i}\epsilon N(x)}K(x_{i}-x)x_{i}}{\sum_{x_{i}\epsilon N(x)}K(x_{i}-x)}$$

where, *N*(x) is the neighborhood of samples within a given distance around x, *m*(x) − x is the mean-shift.

Updating a centroid x to the mean of the samples within its neighborhood *m*(x), i.e., x = *m*(x), and repeat the estimation process until *m*(x) converges. The algorithm automatically sets the number of clusters, and it is guaranteed to converge. The algorithm will stop iterating when the change in centroids is small.

#### 3.1.2. Extract Multifractal Features of Each POR Class

In the actual situation, since the POR evolution curve cannot be characterized by a single dimension, we adopt the multifractal method to achieve the feature description. Multifractal dimension is a measure that uses dimension to describe the characteristics of a fractal set. A method of calculating fractal dimension based on box dimension is as follows:

(1) Cover the one-dimensional POR time series by a box of length δ; that is, divide it into N non-overlapping intervals according to the time scale δ. Then the probability measures are the proportion of the set x falls in each small box to the total, i.e.,

(2) $$P_{i}\left(\delta \right)=\frac{N_{i}}{N},\;i=1,2,\ldots ,N$$

where, *δ* is the length of each box; *N* is the total number of points in the set x, and $N_{i}$ is the number of points in the box containing the *i* th box. 

(2) Define the partition function of a multifractal system:

(3) $$\chi \left(q,\delta \right)=\sum_{i=1}^{N}P_{i}^{q}(\delta )$$

where, $\chi \left(q,\delta \right)$ reflects the uniformity of $P_{i}$, q is the weight factor, and the values of *q* can range from −∞ to +∞, when q>>1, the large probability subset plays the main role in the partition function. For q<<−1, the small probability subset plays the main role in the partition function. The box size δ can be considered as a filter so that big values of the size is equivalent to apply a large-scale filter to the pattern. Changing the size δ, we can explore the sample at different scales. Therefore, the partition function $\chi \left(q,\delta \right)$ provides information at different scales and moments. 

(3) Define the generalized fractal dimension:

(4) $$D(q)=\left\{\begin{array}{c} \frac{1}{q-1}\underset{\delta \to 0}{lim}\frac{log\sum_{i=1}^{N}P_{i}^{q}(\delta )}{log\delta },\;\;\;\;\;\;\;\;\;\;\;\;\;q\neq 1\\ \;\;\;\;\;\underset{\delta \to 0}{lim}\frac{log\sum_{i=1}^{N}P_{i}^{q}(\delta )\times lnP_{i}^{q}(\delta )}{log\delta },\;\;q=1 \end{array}\right..$$

(4) Obtain *D*(*q*) spectrum. The partition function $\chi \left(q,\delta \right)$ and δ have the following power–law relationship:

(5) $$\chi \left(q,\delta \right)\propto \delta^{D(q)}$$

where the slope D(q) can be obtained by log fitting $\chi \left(q,\delta \right)\propto \delta^{D(q)}$ curve. Then change q, the corresponding *D*(*q*) spectrum is obtained by drawing a graph in the *q*-*D*(*q*) space.

#### 3.1.3. Construct the POR Situation Discriminative Model

In order to enhance the discrimination performance of the POR situation, we introduce the SVM classifier to find the best hyperplane in order to optimize the grouping of samples. The hyperplane is found through the maximum margin, i.e., the maximum distance between sample fractal features of classes, so as to achieve the intended purpose of more accurate samples’ classification. 

Suppose the training set is a set of N observations as follows:

{(*x_i_, y_i_*), i = 1, 2, …, N, *x* ⊆ R^N^, *y* ⊆ R }

the vectors x_i_ are the patterns belonging to the input space. The scalars *y_i_* are the labels (targets). In a classification problem we have that *y_i_* ∈ {−1, 1}.

The equation of a hyperplane is as follows:

(6) $$w\varphi \left(x\right)+b=0$$

where *x* = (*x*_1_, *x*_2_, …, *x_d_*) is the input data with dimension d, *w* =(*w*_1_, *w*_2_, …, *w_d_*) is a vector normal to the hyperplane and b is an offset, which determines the distance between the hyperplane and the origin. The distance from any point *x* in the sample space to the hyperplane (*w*, b) can be written as follows:

(7) $$r=\frac{\left|w^{T}\varphi \left(x\right)+b\right|}{\left|\left|w\right|\right|}.$$

Taking the multifractal dimension {*D*(1), *D*(2), … *D*(*d*)} of POR samples from each group as the training sample set. Then, the multifractal dimension of the classified POR situations is used as the input vector to train the SVM classifier, and a non-linear SVM classification algorithm is adopted. Given the input dataset and the learning objective f(*x*) = {*x*_1_, *x*_2_, …, *x_N_*}, g(*y*) = {*y*_1_, *y*_2_,… *y_N_*}, for a given two-class classification problem, a non-linear SVM solves the following convex optimization problem:

(8) ESVM(w,ξi)=min w,b⁡12|w|2+C∑i=1Nξi s. t. yiwTφxi+b≥1−ξiξi=argminf·g∑Nmax(0,l−f(xi)·g(yi)+maxf(xi)·g(yi))

where *w* is the weight vector, *C* is a user-specified positive parameter, the $\xi_{i}$ is called the slack variables, *l* is a custom data, and *N* is the total number of samples. We need to find the optimum values of the weight vector *w*, bias b, and the slack variables $\xi_{i}$ to minimize *E_svm_*. 

The Lagrangian for this problem is given by the following:

(9) $$L\left(w,b,\xi ;\alpha ,\nu \right)=E\left(w,\xi \right)-\sum_{i=1}^{N}\alpha_{i}\left\{y_{i}\left[w^{T}\varphi \left(x_{i}\right)+b\right]-1+\xi_{i}\right\}-\sum_{i=1}^{N}\nu_{i}\xi_{i}$$

with Lagrange multipliers $\alpha_{i}\geq 0$, $\nu_{i}\geq 0$ (*i* = 1, …, N). The solution is characterized by the saddle point of the Lagrangian: $\underset{\alpha ,\nu }{max}\;\underset{w,b,\xi }{min}\;L\left(w,b,\xi ;\alpha ,\nu \right)$.

We obtain the following:

(10) $$\begin{array}{c} \frac{\partial L}{\partial w}=0\;\to w=\sum_{i=1}^{N}\alpha_{i}y_{i}\varphi \left(x_{i}\right)\\ \frac{\partial L}{\partial b}=0\;\to \;\sum_{i=1}^{N}\alpha_{i}y_{i}=0\\ \frac{\partial L}{\partial \xi_{i}}=0\;\to {\;0\leq \alpha }_{i}\leq C,\;i,\ldots ,N \end{array}$$

By replacing *w* in the Lagrangian, the following dual problem is obtained:

(11) $$\underset{\alpha }{\max}\sum_{i=1}^{N}\alpha_{i}-\frac{1}{2}\sum_{i=1}^{N}\sum_{j=1}^{N}\left[\alpha_{i}y_{i}\varphi (x_{i}{)}^{T}\varphi \left(x_{j}\right)y_{i}\alpha_{i}\right]\;s.\;t.\;\sum_{i=1}^{N}\alpha_{i}y_{i}=0,\;0\leq \alpha_{i}\leq C$$

where α_i_ is the Lagrangian multiplier and we can choose a kernel motivated by the Mercer condition

*K*($x_{i},x_{j}$) $=\varphi (x_{i}{)}^{T}\varphi \left(x_{j}\right)$ such that there is no need to compute w and $\varphi \left(x_{i}\right)$.

The final SVM classifier is as follows:

(12) $$y\left(x\right)=sign[\sum_{i=1}^{N}\alpha_{i}y_{i}K\left(x,x_{i}\right)+b]$$

with $\alpha_{i}$ positive real constants and b a real constant that follows from the quadratic programming problem. The non-zero Lagrange multipliers $\alpha_{i}$ are called support values. The corresponding data points are called support vectors and are located close to the decision boundary. These are the data points that contribute to the classifier model.

For kernel SVM, the selection of the kernel function is the key to the classification performance of SVM. There are several choices for the kernel *K*(∙, ∙), e.g., linear kernel, polynomial kernel, Gaussian kernel, multilayer perception kernel, etc. Here, we choose the Gaussian kernel $K\left(x,x_{i}\right)=exp\{-\frac{\left||x-x_{i}\right|{|}^{2}}{2\sigma^{2}}\}$ based on the comparison of experimental results. The Mercer condition holds for all σ values in this case [18]. It can ensure the sample is mapped to an appropriate space, which leads to a better classification effect.

For multiclass problems, the one-versus-the-rest strategy is implemented. That is, for the multiclass classification, the same principle is utilized after breaking down the multiclassification problem into multiple binary classification problems. Firstly, using training samples belonging to the same POR group to train the SVM for POR situation discrimination. Secondly, using the validation sample sets extracted randomly to test the accuracy of the output classification result of the SVM model, and constantly adjusting the input vector until a required accuracy threshold is met. Finally, the POR situation discrimination model supported by the SVM classifier is completed.

### 3.2. Construct a Fractal Interpolation-Based POR Prediction Model

Fractal interpolation is a modern technique to fit and analyze non-stationary data and non-smooth curves. We develop a framework of predicting a POR situation based on fractal interpolation iterative algorithm, which converge to a data generating (original) function for any choice of the scaling factors. When new POR information is captured, we need to firstly judge the potential type of POR trend in terms of the above discriminative model, then to calculate the Hurst exponent of this type of POR evolution using the rescaled range analysis (*R/S*). The Hurst exponent is used to tailor the fractal interpolation to match the original data’s complexity. Finally, we perform interpolate from a set of data using fractal interpolation based on iterated functions systems (IFS). Since IFS are capable of generating fractal and multifractal structures, the inherent complexity of fractal interpolated data is closer to the real-time POR data.

#### 3.2.1. Determine the Hurst Exponent

Hurst first proposed the Hurst exponent calculated by rescaled range analysis (*R/S*) in 1951 for the analysis of long-term time series correlations. The *R/S* method I use is as follows: 

(1) Divide the POR time series into a non-overlapping sub-series of length n on average, *A × n* = *N*. Each sub-series is denoted as *I_a_* (*a* = 1, 2,…, *A*), where each element is labeled $N_{k,a}$ (*k* = 1, 2, …, *n*). Then the mean $e_{a}$ of a sub-series of length n is $e_{a}=\frac{1}{n}\sum_{k=1}^{n}N_{k,a}$,

(2) Calculate the extreme deviation sequence on each sub-series *I_a_*:

(13) $$R_{I_{a}}=\underset{1\leq k\leq n}{\max}\left(X_{k,a}\right)-\underset{1\leq k\leq n}{\min}\left(X_{k,a}\right),a=1,2,\ldots ,\;\;A.$$

The standard deviation sequence is calculated as follows:

(14) $$S_{I_{a}}=\sqrt{\left[\frac{1}{n}\sum_{k=1}^{n}{(N}_{k,a}-e_{a}{)}^{2}\right]}$$

here, $X_{k,a}=\sum_{k=1}^{n}{(N}_{k,a}-e_{a}),k=1,2,\ldots ,n$

(3) Arrive the average *R/S* value of length *n* at:

(15) $${{\left(\frac{R}{S}\right)}_{n}=\frac{1}{A}\sum_{a=1}^{A}R_{I_{a}}/S}_{I_{a}}$$

when *n* is large enough, there is (*R/S*)*_n_∝cn^H^*. Where *H* is the Hurst exponent and *c* is a fixed constant. The index is calculated by the least squares method, with values ranging from 0 to 1.

#### 3.2.2. Construct the Fractal Interpolation-Based POR Situation Predict Model

Barnsley proposed the fractal interpolation algorithm based on fractal collage principle in 1986. For datasets: {(*x_i_, y_i_*): *i* = 0,1,…, *N*}, an iterated function system (IFS) for interpolation could be constructed by using the affine transformation. The affine transformation *w_i_* is as follows:

(16) $$w_{i}{\begin{array}{cc} {}[x & y \end{array}]}^{T}=\left[\begin{array}{cc} a_{i} & b_{i}\\ c_{i} & d_{i} \end{array}\right]{\begin{array}{cc} {}[x & y \end{array}]}^{T}+{\begin{array}{cc} {}[e_{i} & f_{i} \end{array}]}^{T}.$$

For Equation (16), let *b_i_* = 0, the other five constants *a_i_*,* d_i_*,* c_i_*,* e_i_*,* f_i_* meet the following four linear equations:

(17) $$a_{i}x_{0}+e_{i}=x_{i-1}$$

(18) $$a_{i}x_{N}+e_{i}=x_{i}$$

(19) $$c_{i}x_{0}+d_{i}y_{0}+f_{i}=y_{i-1}$$

(20) $$c_{i}x_{N}+d_{i}y_{N}+f_{i}=y_{i}$$

The *d_i_* is a free variable, *d_i_* ∈ [0,1], otherwise the IFS do not converge, and the other four constants of *w_i_* can be expressed as follows:

(21) $$a_{i}=\frac{\left(x_{i}-x_{i-1}\right)}{x_{N}-x_{0}}$$

(22) $$c_{i}=\frac{y_{i}-y_{i-1}}{x_{N}-x_{0}}-\frac{d_{i}(y_{N}-y_{0})}{x_{N}-x_{0}}$$

(23) $$e_{i}=\frac{{x_{N}x}_{i}-{x_{i}x}_{0}}{x_{N}-x_{0}}$$

(24) $$f_{i}=\frac{{x_{N}y}_{i-1}-{y_{i}x}_{0}}{x_{N}-x_{0}}-d_{i}\frac{{x_{N}y}_{0}-{y_{N}x}_{0}}{x_{N}-x_{0}}$$

where *d_i_* is a free variable (that is, the vertical scaling factor) and *d_i_* ∈ [0,1), otherwise the IFS do not converge. It can be seen from Equations (21) to (24) that the selection of *d_i_* has a great influence on the calculation of the other four parameters of the affine transformation, so it is particularly important to accurately estimate *d_i_* for the prediction results.

According to the fractal theory, if $\sum_{i=1}^{N}|d_{i}|>1$, and the interpolation points are not collinear, then the fractal dimension *D* of the attractor of the IFS satisfies the following equation:

(25) $$\sum_{i=1}^{N}|d_{i}|a_{i}^{D-1}=1$$

According to Equation (25), if the POR time series have self-similarity properties, then we can use the fractal box dimension *D* to calculate *d_i_*. That is reasonably simple to obtain the value of *d_i_* using H in terms of the relationship of *D* and *H* [19]: *D* = 2 − *H*

Here, we make the following reasonable assumption: assuming that the value of each vertical scaling factor *d_i_* is equal to the size |*d*|, then,

(26) $$\left|d\right|=\frac{1}{\sum_{i=1}^{N}a_{i}^{1-H}}$$

According to Equation (26), after estimate the parameter *H*, we will obtain the value of d, then the other four iterative parameters can be determined by solving the Equations (21)–(24), the complete IFS will be constructed. Doing fractal interpolation based on the IFS would obtain the prediction of the subsequent POR situation.

## 4. Experimental Results

**Figure 1:**
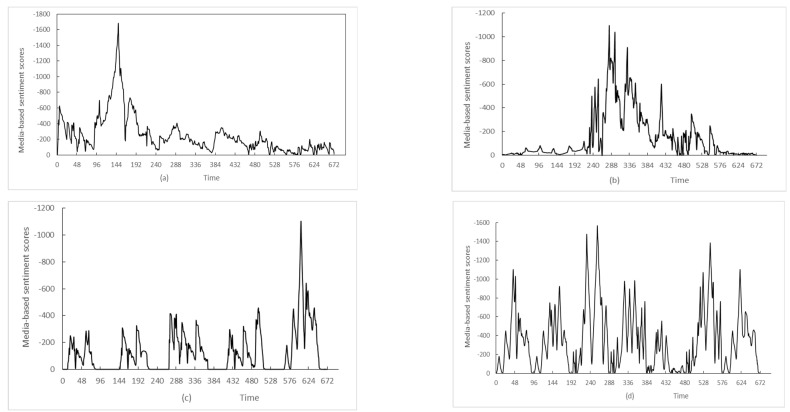
The types of the POR evolution of China’s ODI events (four weeks). Note: (**a**) The upsurge of public opinion appears in early stage; (**b**)The upsurge of public opinion appears in middle stage; (**c**) The upsurge of public opinion appears in later stage; (**d**) The upsurges of public opinion appear repeatedly.

The sample data source of this paper are the media reports of selected 30 host countries who attract the largest amount of China’s ODIs. The countries include Singapore, Indonesia, Malaysia, Thailand, Viet Nam, Pakistan, United Arab Emirates, Cambodia, Serbia, Bangladesh, United States, Germany, Laos, Switzerland, United Kingdom, Russia, South Africa, Spain, Japan, Brazil, Turkey, Italy, New Zealand, Kenya, Uganda, Egypt, India, Guinea, Nigeria, Kuwait, Mauritius, and Tanzania. All relevant media coverage for China’s ODI events in the 30 countries are downloaded from GDELT Event Database from 1 January 2014 to 30 December 2022 (https://analysis.gdeltproject.org/index.html (accessed on 25 January 2023)). Python 3.5.0 is used for pulling the POR data via Google BigQuery. If the tone of the media coverage for China’s ODI events is negative, give it a score −1. Adding the negative tone scores of all media reports referring to an event at a frequency of 1 h/time, we can obtain a POR time series. Figure 1 shows the main four types of POR evolution of China’s ODI events judged by our discriminative model.

The four types of POR of China’s ODI events are, for type (a), the POR is explosive in the early stage, type (b) in the middle stage, type (c) in the later stage, and for type (d), the POR repeats the outbreaks in stages.

**Figure 2:**
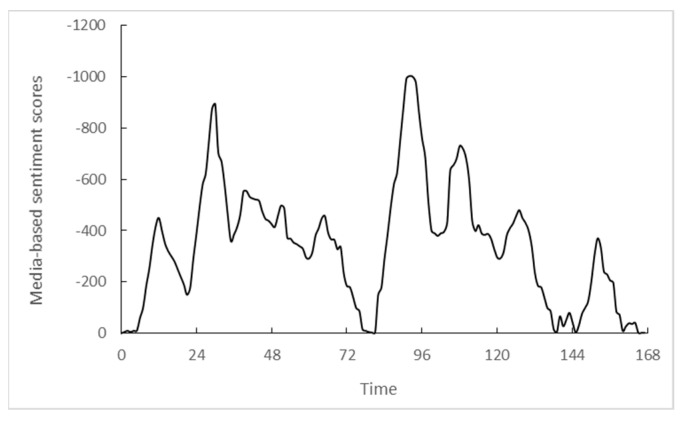
The POR evolution of the tax evasion event of a local arm of China’s MNE in India (one week).

In order to test the validity of the monitoring method developed in this paper, we take the public opinion crisis of the tax evasion incident of the local arm of China’s MNE in India as an example to predict its POR situation. The real-time POR data of “tax evasion event” obtained by crawling website’s pages provided by GDELT Event Database from 5 January 2022 to 2 February 2022. Figure 2 shows the POR outbreak and evolution of the tax evasion event of a local arm of China’s MNE in India. Using the POR situation discriminative model developed in this paper, we can classify the POR situation of “tax evasion event” as type (a) shown in Figure 1.

**Figure 3:**
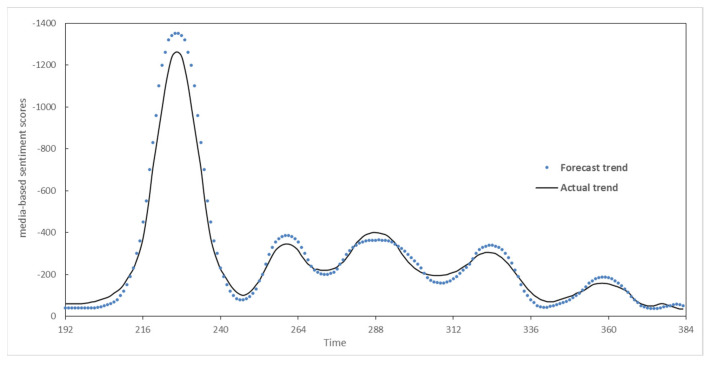
Comparison between predicted results and actual measured.

After we determined the classification of the POR situation of “the tax evasion event”, the POR prediction will be exacted by integrating the real-time POR data into the fractal interpolation-based prediction model. The iterative calculation is carried out through the IFS to predict the subsequent POR propagation situation of “the tax evasion event”. The comparison of some predicted results and actual measured result is shown in Figure 3.

Table 1 gives a comparison between the experimental results and the actual POR evolution of “tax evasion event”. The experimental results show that the POR predicting method based on a multifractal situation optimization described in this paper can predict the POR evolution for different prediction periods with an average accuracy of 92.21% [=(93.62% + 91.57% + 91.44%)/3], and the F_1_ value (the harmonic mean of accuracy and recall rate) reaches 93.03% [=(92.56% + 93.25% + 93.27%)/3], indicating a high predicted accuracy of the POR situation data.

Table 1 shows the results of ablation experiments for different prediction periods. According to whether extracting fractal feature data (multifractal dimension) to train the SVM classifier in the classification stage of the sample dataset, and whether introducing real-time data for interpolation iterative in the prediction stage, there exist the following four strategies for sample datasets processing: no fractal feature data + no real-time data interpolation; fractal feature data + no real-time data interpolation; fractal feature data + real-time data interpolation; And fractal feature data + real-time data interpolation. The following conclusions can be drawn from the ablation results in Table 1:

(1) The historical POR evolutionary trends provide a certain reference for predicting the trend of the similar type of POR events, but it is difficult to achieve a high degree fitting of situation curve;
(2) Using fractal feature data and real-time POR fractal interpolation method can both improve the prediction performance of the new POR situation to a certain extent. The former mainly by refining the subsequent classification results of the POR situation through mining the potential features of curves in different dimensions. The latter is mainly by improving the fit degree of the POR evolution by introducing real-time data interpolation. The prediction accuracy can be maintained with the extension of time and the increase in interpolation points;
(3) Although there exists a bias between the prediction results based on the fractal interpolation algorithm in this paper with the actual measured value, the overall fitting evolutionary trend of POR is consistent with the reality, which proves that this method can improve the prediction accuracy of POR through the processing of the POR data captured.

In order to further verify the performance of the proposed method, the long short-term memory (LSTM) recursive neural network algorithm [21] is used to conduct comparative experiments, and the experimental results are shown in Table 2.

As can be seen from Table 2, the performance of the dynamically monitoring method based on multifractal situation optimization proposed in this paper is superior to the LSTM methods. LSTM needs a very large number of samples to capture the temporal distribution rule of the sample data, but the number of POR events of China’s ODIs is limited. A sample size limitation undermines the reliability of LSTM and reduces its prediction accuracy.

## 5. Conclusions

In view of the increasingly complex media-based POR situation faced by MNEs, this paper proposes a POR monitoring method based on multifractal situation optimization in order to dynamically monitor the POR of ODI event in real-time and help guide the effective control and benign development of public opinion of the event. The fractal characteristics of a POR situation evolution is analyzed with the help of the multifractal dimension theory, and the POR situation discriminative model is built based on the SVM multi-classifier trained with fractal characteristics variables. Then, the fractal interpolation-based prediction model is constructed, using the public real-time POR data incorporated to the IFS can forecast the POR situation with more accuracy and stability, which meets the business needs of real-time dynamic monitoring and early warning of POR very well. 

The outstanding advantages of the POR monitoring method are mainly manifested in the following two aspects: First, different POR guidance and reminders for relevant MNEs and/or authorities can be set according to the various types of discriminating POR situation. For type (a), an early warning signals need to be issued to remind relevant units to monitor the public opinion and take corresponding countermeasures within a short period of time to prevent the rapid spread of negative public opinion. For type (b), the system should pay close attention to monitoring in the early stage and be alert to the possible outbreak of public opinion in the medium term. For type (c), a late warning signals should be issued for such a POR, reminding relevant units not to treat the POR lightly that did not break out in the early stage. And for type (d), a real-time early warning signals should be set up to remind relevant units to pay attention to the evolution of this kind of POR at all times, guiding the actions according to the changing situation. Second, the method could improve the prediction performance of the new POR situation. The usage of multifractal dimensions could refine the classification results of POR situation, and the introduction of real-time POR fractal interpolation method could improve the goodness-of-fit and maintained a high accuracy of prediction with the extension of time and the increase in interpolation points.

## Figures and Tables

**Table 1:** Comparison between the experimental results and the actual POR situation.

Dataset Processing Strategy	Accuracy of Classification	Forecast Period One Week		Forecast Period Two Weeks		Forecast Period Three Weeks	
		Forecast Accuracy (%)	F1 Score (%)	Forecast Accuracy (%)	F1 Score (%)	Forecast Accuracy (%)	F1 Score (%)
Non-fractal feature data + no real-time POR data interpolation	83.62	72.56	70.34	71.22	70.65	69.21	68.35
Fractal feature data + no real-time POR data interpolation	95.88	81.27	75.89	76.23	74.12	71.37	73.28
Non-fractal feature data + real-time POR data interpolation	82.36	82.67	82.78	84.71	83.37	84.26	82.67
Fractal feature data + real-time POR data interpolation	95.88	93.62	92.56	91.57	93.25	91.44	93.27

Note: According to Goutte and Gaussier [20], we formalize the forecast accuracy for class POR situation and F1 score: forecast accuracy = (true positive + true negative)/(true positive + false positive + true negative + false negative); F1 = 2 × precision × recall/(precision + recall), precision = true positive/(true positive + false positive), recall = true positive/(true positive + false negative).

**Table 2:** The experimental results comparison of LSTM with multifractal situation optimization.

Method	Forecast Accuracy (%)	F_1_ Value (%)
LSTM	80.01	76.35
Multifractal situation optimization	95.17	96.23

Note: According to Goutte and Gaussier [20], we formalize the forecast accuracy for class POR situation and F1 score: forecast accuracy = (true positive + true negative)/(true positive + false positive + true negative + false negative); F1 = 2 × precision × recall/(precision + recall), precision = true positive/(true positive + false positive), recall = true positive/(true positive + false negative).

## Data Availability

The POR data are collected from GDELT Event Database at https://www.gdeltproject.org/ (accessed on 25 January 2023).
